# Incidence, root cause, and outcomes of unintentionally retained intraabdominal surgical sponges: a retrospective case series from two hospitals in Togo

**DOI:** 10.1186/s13037-017-0140-2

**Published:** 2017-10-26

**Authors:** Boyodi Tchangai, Mazamaesso Tchaou, Iroukora Kassegne, Kpatekana Simlawo

**Affiliations:** 1 Department of Surgery, Teaching Hospital of Sylvanus Olympio, Lomé, Togo; 2 Department of Radiology, Teaching Hospital of Sylvanus Olympio, Lomé, Togo; 3 General surgery, Lomé Commune Regional Hospital, Lomé, Togo; 4 Visceral surgeon at Sylvanus Olympio Teaching Hospital, 198 rue de la santé, P.O Box 57, Lomé, Togo

**Keywords:** Gossypiboma, Foreign body reaction, Complications, CT scan, Laparotomy, Prevention

## Abstract

**Background:**

The term gossypiboma refers to a sponge that has been forgotten in the surgical field. It is the most common retained surgical item, and constitutes a continuing problem for surgical safety. We performed a hospital-based study to examine their incidence, root cause, and outcomes, as an effort toward improving prevention.

**Methods:**

This retrospective study covered 10 years (2006-2015) and included surgically confirmed cases of abdominal gossypibomas occurring after 45,011 abdominal and gynaecological operations in 2 public hospitals in Lome (Togo). Age, diagnosis, initial surgical procedure, evidence of textile count, and data related to the revision procedure were collected for descriptive analysis.

**Results:**

Fifteen cases of gossypibomas (11 women and 4 men) were recorded. The mean age of the patients was 27 (range 21-55) years. Initial procedures were gynaecological in 11 patients and 5 cases involved an emergency surgery. Evidence of sponge counting was found in 6cases. Gossypiboma was an incidental finding in 1 patient. The average time to onset of symptoms after the initial procedure was 2 months. The gossypiboma was removed within 7 days to 4 years after the initial procedure. Postoperative complications included enterocutaneous fistula in 2 patients, incisional hernia in 2 patients, and wound sepsis in 1 patient. Death occurred in 2 patients (13.3%).

**Conclusions:**

Although rare, the incidence of gossypibomas is still unacceptably high and reveals failures regarding patient safety standards. The associated morbidity and mortality are significant, yet can be reduced by an early diagnosis in the immediate postoperative period. A systematic methodical count of sponges is the cornerstone of prevention, and introducing surgical safety protocols, such as the WHO Safe Surgery Saves Lives checklist, can enhance effectiveness. There is a crucial need for safety-focused policies, which may include a never event reporting system, elaboration of prevention strategies, interventions, and evaluation.

## Background

The term gossypiboma refers to a sponge that has been forgotten in the surgical field [1]. “Gossypiboma” is derived from “gossypium,” which is Latin for cotton, and “boma” which is Swahili for a concealed mass after surgery [2]. Gossypibomas are the most common retained surgical item (RSI); needles, forceps, and retractors may also be RSIs [1, 3]. The highest rate of this medical error occurs in abdominal surgery [2, 4]. Clinical consequences can be dramatic, leading to a high rate of morbidity. The time to diagnosis is increased by a poor knowledge of the circumstances surrounding the occurrence of these accidents, somehow considered to be the exclusive responsibility of the negligent surgeon. RSIs are top-listed as a never medical event, or more appropriately a serious adverse event (Table 1), as defined by the National Quality Forum (NQF) [5]. Like most of the items on this list, gossypibomas are preventable, and thus, are obviously considered as unacceptable errors. Strategies to reduce never event risks include the use of surgical checklists and safety protocols [6, 7]. Despite progress in patient safety policies, these errors have not been eradicated and are still a matter of concern for surgical teams [8]. The actual extent of the problem is unknown, as the reported incidence is thought to reflect just the tip of an iceberg because of underreporting [1]. Achieving the goal of quality improvement and reduction of never events, such as gossypibomas, obviously begins with reporting these defined events [5]. The aim of the present hospital-based study is to examine, the incidence, root cause, and outcomes of gossypibomas, in an effort to contribute to improving prevention.

**Table 1:** Summary of serious reportable events of the National Quality Forum

1. Surgery performed on the wrong body part.
2. Surgery performed on the wrong patient.
3. Wrong surgical procedure performed on a patient.
4. Unintended retention of a foreign object in a patient after surgery or other procedure.
5. Intraoperative or immediate postoperative death in an ASA class I patient.
6. Patient death or serious disability associated with the use of contaminated drugs, devices, or biologics provided by the healthcare facility.
7. Patient death or serious disability associated with the use or function of a device in patient care in which the device is used or functions other than as intended.
8. Patient death or serious disability associated with an intravascular air embolism that occurs while being cared for in a healthcare facility.
9. Infant discharged to the wrong person.
10. Patient death or serious disability associated with patient elopement (disappearance).
11. Patient suicide, or attempted suicide, resulting in serious disability while being cared for in a healthcare facility.
12. Patient death or serious disability associated with a medication error.
13. Patient death or serious disability associated with a haemolytic reaction due to the administration of ABO/HLA-incompatible blood or blood products.
14. Maternal death or serious disability associated with labour or delivery in a low-risk pregnancy while being cared for in a healthcare facility.
15. Patient death or serious disability associated with hypoglycaemia, the onset of which occurs while the patient is being cared for in a health care facility.
16. Death or serious disability (kernicterus) associated with failure to identify and treat hyperbilirubinemia in neonates.
17. Stage 3 or 4 pressure ulcers acquired after admission to a healthcare facility.
18. Patient death or serious disability due to spinal manipulative therapy.
19. Artificial insemination with wrong donor sperm or wrong egg.
20. Patient death or serious disability associated with an electric shock while being cared for in a healthcare facility.
21. Any incident in which a line designated for oxygen or other gas to be delivered to a patient contains the wrong gas or is contaminated with toxic substances.
22. Patient death or serious disability associated with a burn incurred from any source while being cared for in a healthcare facility.
23. Patient death or serious disability associated with a fall while being cared for in a healthcare facility.
24. Patient death or serious disability associated with the use of restraints or bedrails while being cared for in a healthcare facility.
25. Any instance of care ordered by or provided by someone impersonating a physician, nurse, pharmacist, or other licensed healthcare provider.
26. Abduction of a patient of any age.
27. Sexual assault on a patient within or on the grounds of a healthcare facility.
28. Death or significant injury of a patient or staff member resulting from a physical assault (i.e. battery) that occurs within or on the grounds of a healthcare facility.

## Methods

This retrospective study covered 10 years (from January 1, 2006 to December 31, 2015) and included surgically confirmed cases of abdominal gossypibomas in the general/visceral surgery and gynaecological departments of two hospitals in Lome, Togo, West Africa (Lome Commune regional hospital and Sylvanus Olympio teaching hospital). These two hospitals are the only surgical public facilities serving the urban demographic area of Lome.

During the study period, 45,011 abdominal and pelvic procedures (15,790 in Lome Commune regional hospital and 29,221 in Sylvanus Olympio teaching hospital) were performed, including those in the gynaecological departments. At the time of the study, the two hospitals did not have an institutional policy regarding quality improvement, especially in the surgical safety area, and the WHO surgical checklist, or any surgical safety checklist, was not implemented. Furthermore, there were no institution-level specific protocols to reduce the incidence of never events, including RSIs. Although a sponge count was recommended, no standardized count practice was established. Radiopaque sponges were not always available, and the hospitals did not have specific equipment for an intra-operative X-ray search of a forgotten sponge.

Using the operative registries, summaries of all gynaecological and abdominal procedures during the study period, including name, date, operative diagnosis, and procedure, were manually reviewed, as there was no coding system, or a specific reporting procedure. Reviewers were asked to select, for a thorough screening of the surgery reports, patients with the following terms indicated in the diagnosis box: foreign body, gossypiboma, and swab. Additionally, the surgeons and gynaecologists at the 2 hospitals, as well as others involved in the identified cases, were interviewed in order to complete data collection from the patient’s records. The data were reported on a form created in EPI Info 7 for descriptive analysis.

And concerned the following parameters: age, diagnosis, initial surgical procedure, evidence of textile count, and data related to the revision procedure (clinical sign leading to the diagnosis of gossypiboma, imaging results, operative findings, surgical treatment, and outcomes).

## Results

Fifteen cases of gossypibomas (11 women and 4 men; sex ratio, 0.36) were recorded in the two hospitals. The overall incidence was 1/3030 procedures, with an incidence of 1/2656 (4 cases) and 1/3947 (11 cases) at the Lome Commune regional hospital and Sylvanus Olympio teaching hospital, respectively. The mean age of the patients was 27 (range, 21-55) years. Table 2 provides the indications of the 15 initial procedures of which 7 were made in an emergency setting. Operations were carried out through a transverse supra-pubic laparotomy in 10 cases, a median laparotomy in 4 cases, and an inguinal incision in 1 case. The initial operation involved a different operator surgeon in each case. Operator was a confirmed surgeon in 11 cases and a training surgeon in 4 cases. Evidence of sponge counting was found in 6 operative records.

**Table 2:** Indications of the initial operations of patients with gossypiboma

	Indications (n)
Hysterectomy (5)	Uterine myomas (3), uterine perforation (2)
Myomectomy (4)	Primary infertility (3)
Caesarean section (2)	Foetal distress (2)
Intestinal resection (1)	Acute intestinal obstruction
Left colectomy (1)	Cancerous colic obstruction (1)
Mac Vay repair (1)	Femoral hernia (1)
Splenectomy (1)	Blunt abdominal trauma (1)

**Fig. 1:**
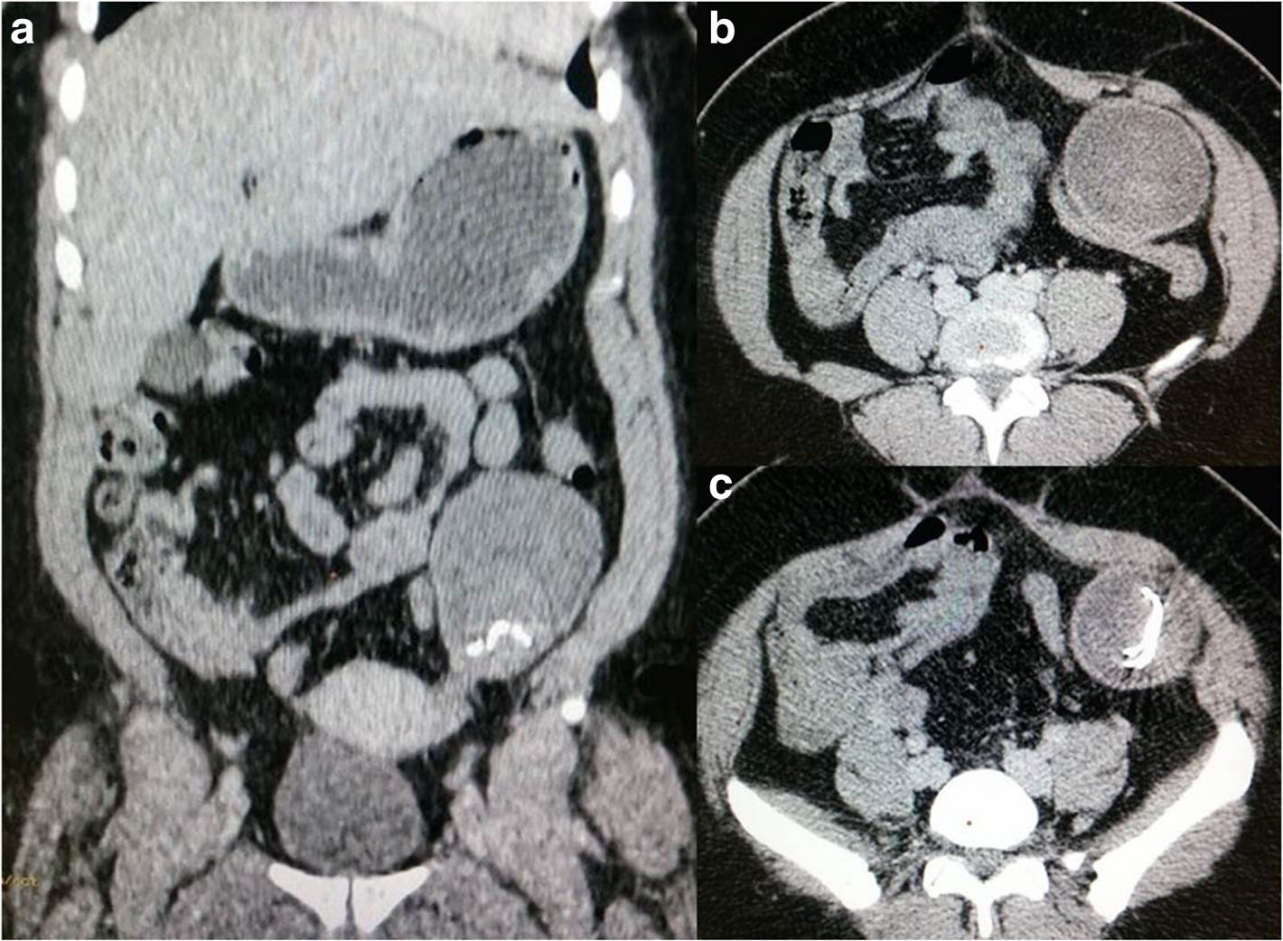
Cornal (**a**) and axial (**b** and **c**) abdominal CT scan images showingan organised hypodense mass (gossypiboma) in the left iliac fossa with an intern hyperdense structure corresponding to a radiopaque marker

**Fig. 2:**
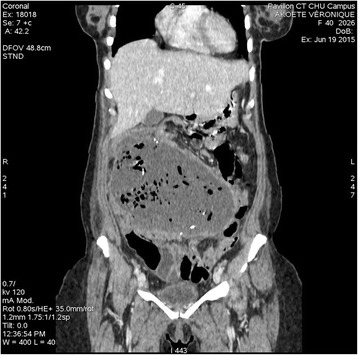
Cornal abdominal CT scan image;large well limited hypodense mass (gossypiboma) withaeric content and rares calcifications

**Fig. 3:**
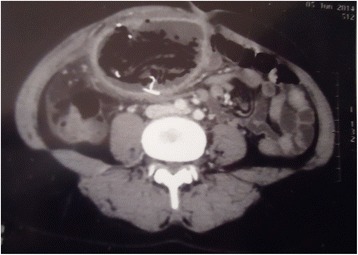
Abdominal CT scan; well limited hypogastric mass (gossypiboma) with hydro aeric content and rares calicifications andhyperdense structure coresponding to a radiopaque marquer

**Fig. 4:**
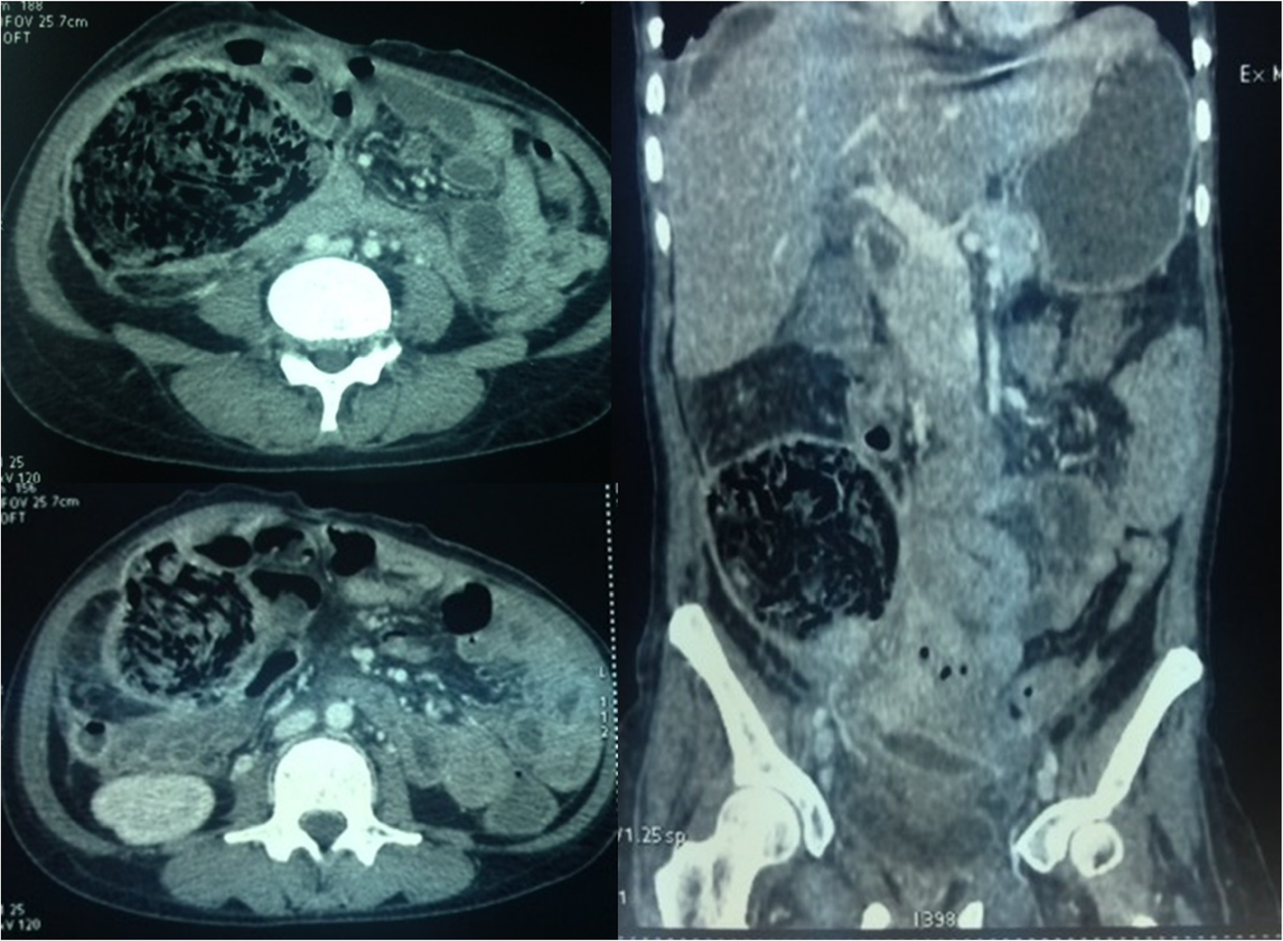
Axial (**a** and **b**) and coronal (**c**) abdominal CT scan images showingan heterogenous mass (gossypiboma) in the right flanc presenting a spongiform pattern

Gossypiboma was an incidental finding in 1 patient during an elective re-operation. Signs presented by other patients are shown in Table 3. The average time to onset of symptoms after the initial surgery was 2 months (range 5 days to 7 months). Plain abdominal radiography was performed in 3 patients, revealing hydroaeric levels in 2cases, and no abnormalities in 1 case. Abdominal ultrasonography was performed in 8 patients and was contributory in 5 patients, showing a hypoechoic (3 patients) or heterogeneous (2 patients) well circumscribed mass. An abdominopelvic -enhanced computed tomography (CT) scan was performed in 5 patients (Figs. 1, 2, 3 and 4). A well-circumscribed mass with enhanced wall was observed in all the cases. The mass was hypodense with rare calcifications and aeric content in 3 cases; and heterogeneous with a spongiform pattern in 2 cases. A hyperdense linear image corresponding to the opaque radio-marker of the textile was found within the mass in 2 cases. The preoperative diagnosis workup suspected gossypiboma in 6 cases, a tumour in 3 cases, a fecaloma in 1 case and a postoperative abscess in 4 cases.

**Table 3:** Distribution of clinical signs in 15 patients with gossypiboma

	Number
Abdominal pain	13
Fever	6
Cessation of flatus and bowel movement	5
Abdominal tenderness	4
Vomiting	3
Wound sinus	3
Abdominal mass	2
Enterocutaneous fistula	2

**Fig. 5:**
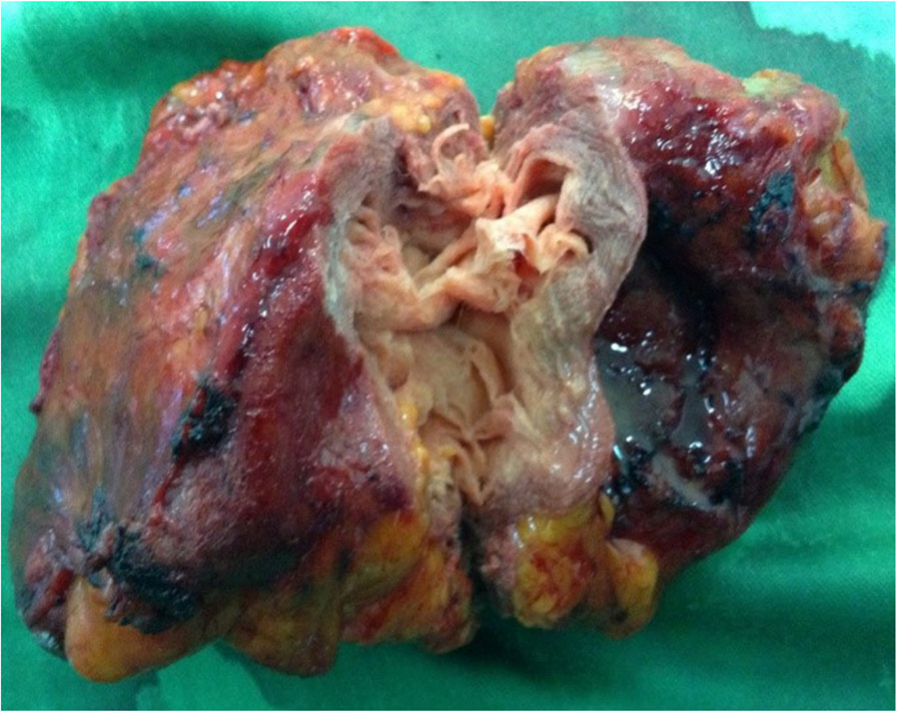
Excised abdominal gossypiboma presenting as a pseudo-tumour fibrous reaction

The median date of detection (by surgery or preoperative workup) was 9 months after surgery (range, 7 days to 4 years after surgery). The gossypiboma was removed via a midline laparotomy in 14 patients and an inguinal incision in 1 patient. The removal was performed in 8 patients within the first year after the initial procedure. The gossypiboma was a small sponge (10×10cm) in 3 cases and a large laparotomy sponge (30×30cm) in 12 cases. The macroscopic appearance of the gossypiboma corresponded to a pseudo-tumour fibrous reaction in 3cases (Fig. 5). There were adhesions involving the abdominal wall in 2 cases, the intestine in 10 cases, and the colon in 2 cases. There was a purulent collection surrounding the sponge in 9 cases. Two cases of intestinal migration were found, one of which was completely sealed (absence of fistula and peritonitis) and the other accompanied by enteric fistula and peritonitis. Intestinal resection was performed in 11 cases followed by anastomosis in 10 cases and stoma in 1 case. Postoperative morbidity was observed in 5 patients: enterocutaneous fistula in 2 patients, incisional hernia in 2 patients, and wound sepsis in 1 patient. The enterocutaneous fistula spontaneously dried up in 1 patient. Death occurred in 2 patients (13.3%): one died on the second postoperative day of a sepsis that appeared prior to reoperation and the other died on the ninth postoperative day after the recurrence of an enterocutaneous fistula. In those patients, removal of the gossypiboma took place in the second and third month after the initial procedure, respectively. The presence of the gossypiboma was not disclosed to the patient in 3 cases. A claim was initiated in 2 cases, leading to compensation in both cases.

## Discussion

Gossypibomas are the most common RSI [1, 3]. Their frequency after abdominal surgery has been estimated in previous reports to be between 1/1000 and 1/1500 operations [9, 10]. However, this must be considered as an underestimation, as many reasons can affect their reporting, including medicolegal concerns [1, 3, 8, 9] Gossypibomas are recognised as a medical never event and may be medicolegally indefensible according to the doctrine, res ipsa loquitur [11]. Moreover, they constitute objective evidence of the quality of care and must remain exceptional. The incidence found in the present study is high compared to that in a recent nationwide study conducted in the US (1/7692 abdominal operations) [12] and another study based on a large insurance administrative dataset (incidence of all RSI, 1/8801 to 1/18,760 inpatient operations) [3]. The incidence in the present study is less than that reported in similar low-income settings (1/833 in a study by conducted in Senegal [13] and 1/769 in a study conducted in the Ivory Coast [14]). However, no strong conclusions can be drawn from these comparisons, considering the discrepancies in the incidence denominator and bias arising from case recruitment. The incidence in the present study should be considered unacceptably high in any health care standards.

Risk factors of gossypibomas that have been reported in the literature include emergency surgery, high body mass index, unplanned change in the type of surgery, and a complex surgical procedure [1, 3, 4]. As in other studies [15, 16], we noted a higher proportion of women and gynaecological interventions. Supra-pubic transverse laparotomy, which is commonly used during these procedures, lacks good abdominal exposure, thus increasing the risk of gossypiboma.

Nearly half of the gossypibomas were found more than a year after the initial operation in our study. Although some patients may remain totally asymptomatic for a long time [17], it is a clue that there is a weak index of suspicion of gossypibomas in patients presenting with postoperative abdominal symptoms. Clinical signs depend on the type of immunological foreign body reaction and on bacterial colonisation [8, 10]. In cases with an exudative reaction, there are more acute signs of sepsis [16], as observed in several of our patients with abscess and peritonitis. In contrast, symptoms are subacute (transit disorder, mass, non-specific abdominal pain) when the reaction is essentially fibro-granulomatous [16, 18]. The size of the sponge may also play a role in the clinical manifestation [4], which may explain why most of the retrieved textiles were large laparotomy sponges. Patients may also present with rarer complications such as enterocutaneous fistula and intestinal migration of the gossypiboma [8, 19, 20]. Imaging is important for preoperative diagnosis. Plain abdominal radiography can easily reveal the diagnosis if the swabs contains a radiopaque marker, unless it has degraded [8, 21]. Abdominal ultrasonography, when not hindered by intestinal gas, may typically elicit an echoic mass with hyper-echogenic internal structures and a posterior shadow [16, 21]. In the present study, we more frequently found hypoechoic cystic-like images, which are also part of the ultrasound semiology of gossypibomas [22]. Differential diagnosis with a postoperative collection is more difficult in this case. Enhanced CT is the most useful imaging test to detect gossypibomas [16]. In many reports, a well-limited hypodense mass with a spongiform pattern and central heterogeneous density including calcifications is its most typical aspect [23, 24], which we found in 2 out of 5 cases. The unique hyperdense display of a radiopaque marker can be useful in less typical cystic like images. Despite an imaging workup, a differential diagnosis considering a fecaloma, tumour, and postoperative abscess may be difficult to achieve [25]. If there is any doubt, surgical exploration is mandatory.

The surgical treatment of gossypibomas is unequivocal [8, 10, 19] and can be avoided only in exceptional cases of transmural migration and expulsion by natural orifices [26]. Open surgery seems safer than laparoscopy, described by some authors [16], which may be used only in selected cases. Most of the gossypibomas that we found involved adhesions with the abdominal wall or with the intestinal loops. Their excision is not always a simple procedure, and the surgical difficulty may be increased by the presence of a complication [8]. Morbidity associated with gossypibomas and their removal was high in our study, with enteric fistula and sepsis being the most threatening conditions. Although most cases are treated successfully, mortality in previous studies [2, 19], as in 2 cases in the present study, highlights the unpredictable course of gossypibomas. Prevention should be further stressed more to mitigate the gossypibomas problem, beginning by systematic evaluation of their occurrence. The present study shows that the sponge count is a critical failure mode that needs to be addressed, as it was not performed in more than half of the cases and was inaccurate in the remaining cases. The correct count of sponges as a systematic and methodical process is the central element in effective prevention [27], although this might be not sufficient to completely eradicate the risk of RSIs [28]. The occurrence of gossypibomas in the present setting raise the more global issue of surgery-focused quality of care policies and their implementation to reduce never events. Problems related to the inaccuracy and absence of a count in the present institutions may be symptomatic of the lack of surgical safety protocols. Among the available surgical safety protocols, the WHO checklist has strong evidence supporting its preventive effect in a wide range of settings [29]. Its adoption and implementation could strengthen the systematisation of sponge counting. In addition, it provides an effective tool to introduce the communication skills and patient safety culture that are necessary to develop positive attitudes necessary to prevent never events, including gossypibomas [30]. Effort must be made along this path, particularly in low-income settings. However, sustainable implementation in such settings must address specific issues, including adaption to suit to local practices, training, and decreasing motivation over time [31]. Other inexpensive measures include a systematic revision of the surgical site before cavity closure and exclusive use of radiolucent sponges. Advanced tagging systems and systematic postoperative radiography have been proposed as a second layer of preventive measures [1, 8]. However, they cannot be applied in the present context, as their cost varies from $95,000 to $1.4 million per prevented retained surgical sponge [28].

## Conclusions

Although rare, the incidence of gossypibomas is still unacceptably high regarding patient safety standards toward never events. They are evidence of failures in preserving the quality of care and safety around surgical cares in the present low-income setting. Morbidity and mortality associated with gossypibomas are high, yet can be significantly reduced by an early diagnosis in the immediate postoperative period. A systematic methodical sponge count is the cornerstone of prevention. Introducing surgical safety protocols, such as the WHO checklist, can be a part of focused policies to improve global surgical safety and prevent never events, including gossypibomas. A reporting system of these medical errors remains necessary to provide valuable insights for the elaboration of prevention strategies, interventions, and evaluation.
